# Hepatic stellate cells contribute to liver regeneration through galectins in hepatic stem cell niche

**DOI:** 10.1186/s13287-020-01942-x

**Published:** 2020-09-29

**Authors:** Jian-Yun Ge, Yun-Wen Zheng, Tomonori Tsuchida, Kinji Furuya, Hiroko Isoda, Hideki Taniguchi, Nobuhiro Ohkohchi, Tatsuya Oda

**Affiliations:** 1 Department of Gastrointestinal and Hepato-Biliary-Pancreatic Surgery, Faculty of Medicine, University of Tsukuba, Tennodai 1-1-1, Tsukuba, Ibaraki 305-8575 Japan; 2 Department of Regenerative Medicine, School of Medicine, Yokohama City University, Yokohama, Kanagawa 236-0004 Japan; 3 Institute of Regenerative Medicine and Affiliated Hospital, Jiangsu University, Zhenjiang, 212001 Jiangsu China; 4 Division of Regenerative Medicine, Center for Stem Cell Biology and Regenerative Medicine, The Institute of Medical Science, The University of Tokyo, Tokyo, 108-8639 Japan; 5 School of Biotechnology and Heath Sciences, Wuyi University, Jiangmen, 529020 Guangdong China; 6 Faculty of Life and Environmental Sciences, University of Tsukuba, Tsukuba, Ibaraki 305-8572 Japan

**Keywords:** Galectins, Hepatic stem cell niche, Hepatic progenitor cells, Hepatic oval cells, Hepatic stellate cells, Liver regeneration, Partial hepatectomy

## Abstract

**Background:**

As a critical cellular component in the hepatic stem cell niche, hepatic stellate cells (HSCs) play critical roles in regulating the expansion of hepatic stem cells, liver regeneration, and fibrogenesis. However, the signaling of HSCs, particularly that involved in promoting hepatic stem cell expansion, remains unclear. While the overexpression of galectins has been identified in regenerating liver tissues, their involvement in cell-cell interactions between HSCs and hepatic stem cells remains to be elucidated.

**Methods:**

To generate a liver regeneration rat model and establish a hepatic oval cell microenvironment as a stem cell niche, 2-acetylaminofluorene treatment plus partial hepatectomy was performed. Immunofluorescence staining was conducted to detect the emergence of hepatic stem cells and their niche. Liver parenchymal cells, non-parenchymal cells, and HSCs were isolated for gene and protein expression analysis by qPCR or western blotting. To evaluate the effect of galectins on the colony-forming efficiency of hepatic stem cells, c-Kit^−^CD29^+^CD49f^+/low^CD45^−^Ter-119^−^ cells were cultured with recombinant galectin protein, galectin antibody, galectin-producing HSCs, and galectin-knockdown HSCs.

**Results:**

Following liver injury, the cytokeratin 19^+^ ductal cells were robustly induced together with the emergence of OV6^+^CD44^+^CD133^+^EpCAM^+^ hepatic stem cells. The activated desmin^+^ HSCs were recruited around the periportal area and markedly enriched in the galectin-positive domain compared to the other non-parenchymal cells. Notably, the HSC fraction isolated from regenerating liver was accompanied by dramatically elevated gene and protein expression of galectins. Hepatic stem cells co-cultured with HSCs significantly enhanced colony-forming efficiency. Conversely, single or double knockdown of galectin-1 and galectin-3 led into a significant function loss, impaired the co-cultured hepatic stem cells to attenuated colony size, inhibited colony frequency, and reduced total cell numbers in colonies. On the other hand, the promotive function of galectins was further confirmed by recombinant galectin protein supplementation and galectins blocking antibodies.

**Conclusions:**

Our findings, for the first time, demonstrated that galectins from activated HSCs contribute to hepatic stem cell expansion during liver regeneration, suggesting that galectins serve as important stem cell niche components.

## Background

The liver has a unique and tremendous capability for regeneration. Following partial hepatectomy, liver mass complete reconstitution was achieved within weeks in rodents [1, 2]. In humans, liver regeneration is most pronounced within the first 2 weeks post-surgery, with the liver mass expansion ranging from 21 to 60% [3–5]. This remarkable regenerative capability is attributed mainly to the replication potential of mature hepatocytes and cholangiocytes. However, in chronic liver disease or acute severe injury, the replicative potential of these mature cells is impaired. As a compensation, the hepatic stem/progenitor cells (HSPCs), which have the potential to differentiate either into hepatocytes or biliary epithelial cells, are induced to replenish the cellular loss [6, 7].

The HSPCs emerge along with an associated niche composed of other resident cellular components, including hepatic stellate cells (HSCs)/myofibroblasts, macrophages, endothelial cells, and the extracellular matrix (ECM), which all contribute to sustaining and modulating liver regeneration activity [7]. Cell signaling from stellate cells and macrophages within the niche reportedly influences the proliferation, migration, and differentiation of HSPCs [8–10], while the close association of matrix signaling, especially laminin, with the expansion and differentiation fate of HSPCs was recently uncovered [11]. The identification of unknown hepatic stem cell niche components and their specific functions would aid in the comprehensive understanding of the signaling network during liver regeneration and disease progression.

Galectins belong to the β-galactoside-binding lectin family, which contains carbohydrate recognition domains that are highly conserved. By cross-linking to cell surface glycoconjugates, galectins regulate diverse biological functions such as cell cycling, migration, ECM remodeling, cell-cell and cell-matrix interactions, angiogenesis, immunity, and inflammation [12]. Among the galectin family, galectin-1 and -3 were reported particularly important in multistep tumor initiation, progression, and metastasis [13–15], thus drawing considerable attention. In the past two decades, galectin-1 and -3 activation in liver fibrogenesis and hepatocellular carcinoma had emphasized their roles in the liver microenvironment [16–18]. Interestingly, galectins also engaged in liver development early during embryogenesis [19, 20]. Recently, their roles in regulating liver regeneration have gradually been uncovered [21, 22].

HSCs are the major source of ECM in the liver [23] and were found capable of generating galectins for their self-activation and growth promotion in the fibrotic liver [24]. Despite the strong association between HSC activation and liver regeneration [25–27], to date, no study has examined the roles of galectins in modulating signaling interactions between HSCs and HSPCs within the niche during liver regeneration. In this study, hepatic stem cell niche was modeled in a rat model following liver injury-induced hepatic oval cell response to identify the role of galectins in this specific microenvironment, especially in mediating the promotive effect of HSCs on hepatic stem cell expansion.

## Methods

### Animals

Eight-week-old male Fischer 344/N Slc rats and embryonic day 13.5 C57BL/6 J mice were purchased from Japan SLC (Shizuoka, Japan). The rats were maintained (two per cage) under standard conditions (22 °C; 50% humidity; and light/dark cycle of 12 h), with free access to food and water for 5 days before the experiments. The mice were subjected to hepatic stem cell isolation immediately upon arrival (see details below). A statement on ethics approval for animal studies is included in the declaration sections.

### Liver regeneration model

Rats were randomized into two groups, including the control (*n* = 6) and model (*n* = 8) groups. The rat model of liver regeneration was established, as we reported previously [28]. Briefly, rats were fed 2-acetylaminofluorene (2-AAF; Wako Pure Chemical Industries, Osaka, Japan) in the diet at a dose of 12 mg/kg body weight per day. After 1 week, 70% of partial hepatectomy was performed under isoflurane (Pfizer Japan Inc., Tokyo, Japan) anesthesia, with the removal of median and left liver lobes.

### Isolation of liver parenchymal and non-parenchymal cells

At weeks 0, 1, 2, 3, 4, and 6 after 2-AAF/PH treatment, the rats were subjected to a standard two-step collagenase perfusion for isolation of liver parenchymal and non-parenchymal fractions following a published protocol [29]. Briefly, the rat was anesthetized and cannulated via the portal vein, and the liver was perfused with buffer containing 0.5 mM thylene glycol tetraacetic acid (EGTA; Wako), followed by perfusion solution containing 0.5 mg/ml collagenase (Worthington Biochemical, Lakewood, NJ, United States). Following perfusion for 20 min, the cells were detached by gentle shaking of the liver and dispersed into Williams’ Medium E (Gibco, Grand Island, NY, USA) containing 10% fetal bovine serum (FBS; Gibco). The suspended cells were collected and filtered through gauze and then washed three times, followed by centrifugation at 50×*g* for 2 min. The cell pellets were collected as parenchymal cells (PCs), and the supernatants were obtained as non-parenchymal cells (NPCs).

### HSCs purification and culture

HSCs were isolated from NPCs using a previously reported method [30]. Briefly, the NPCs supernatants were centrifuged at 450×*g* for 10 min. After which, the cell pellet was collected and centrifuged on an 8.2% Nycodenz cushion (Sigma-Aldrich, St. Louis, MO, USA) at 1400×*g* for 15 min. Subsequent centrifugation of the cells in the upper layer generated the cell pellet enriched with HSCs which was then washed in culture medium containing Dulbecco’s modified Eagle’s medium (DMEM; Thermo Fisher Scientific, Waltham, MA, USA), 10% FBS, and 100 U penicillin/streptomycin (Gibco). The purified HSCs were resuspended in the culture medium and seeded onto a 10-cm tissue culture dish. The cells were cultured at 37 °C in an incubator with 50 ml/L CO_2_. The medium was changed at 24 h after seeding and every other day following until the cells reached 80% confluence.

### Hepatic stem cells sorting

Mouse hepatic stem cells were sorted from the liver of embryonic day 13.5 C57BL/6 fetal mice (*n* = 4), as we described previously [31]. In brief, liver cells were stained with the following antibodies: biotinylated anti-Ter-119 and anti-CD45, anti-c-Kit-APC, anti-CD49f-PE, and anti-CD29-FITC. Streptavidin-labeled allophycocyanin-Cy7 was used to detect biotinylated antibodies. All the above antibodies were purchased from BD Pharmingen, San Diego, CA, USA. For gating, the CD45^−^Ter119^−^ c-Kit^−^ cell population was firstly gated out, and then the CD49f^+/low^CD29^+^ subpopulation was set as the sorting gate. Analysis and sorting were performed using MoFlo with Summit version 4.0 software (DakoCytomation, Denmark).

### siRNA transfection

Cells were transfected with siRNA for galectin-1 (ON-TARGETplus SMARTpool, L-090699-02-0005), siRNA for galectin-3 (ON-TARGETplus SMARTpool, L-087975-02-0005), and siRNA for negative control (ON-TARGETplus Non-targeting pool, D-001810-10-05). All the above siRNAs were purchased from Dharmacon, Lafayette, CO, USA. Transfection with Lipofectamine RNAiMAX (Thermo Fisher Scientific) was performed according to the manufacturer’s instructions. Cells were used for experiments 72 h after siRNA transfection.

### Co-culture and clonal colony assay in vitro

One day before co-culture, the HSCs treated with or without galectin-1 and/or galectin-3 siRNA were seeded at a density of 5 × 10^4^ cells/well onto the membrane of transwell inserts (0.4 μm pore size; Corning, NY, USA) and allowed to attach in DMEM containing 10% FBS. On the same day, the hepatic stem cells were seeded on the bottom of a type IV collagen (Corning) pre-coated 6-well plate at a density of 30 cells/cm^2^ in the presence of 10% FBS, or 2000 cells/cm^2^ with 1% FBS. Twenty-four hours later, the transwell inserts were put into the plate to set up the co-culture. The culture medium used for co-culture consisted of Williams’ Medium E (Gibco), 1% or 10% FBS, 1 μg/ml insulin (Wako), 2 mmol/L L-glutamine (Gibco), 50 mmol/L HEPES (Wako), 10 mmol/L nicotinamide (Sigma), 1 × 10^− 7^ M dexamethasone (Sigma), 20 ng/ml epidermal growth factor (EGF; Sigma), 20 ng/ml hepatocyte growth factor (HGF; Sigma), 50 mmol/L β-mercaptoethanol (Sigma), and 100 U penicillin/streptomycin (Gibco). Cells were cultured at 37 °C in a humidified atmosphere containing 50 ml/L CO_2_. In some cases, blocking antibodies against mouse galectin-1 (R&D Systems, Minneapolis, MN, USA) and galectin-3 (R&D) were added in the culture at a concentration of 0, 2, 4, 16 μg/ml. In other cases, a recombinant mouse galectin-1 (R&D) was added at a concentration of 3, 8, 15, 30, and 40 μg/ml. The culture medium was changed every 2 days, with or without the addition of the above supplements. The number and size (cell number per colony) of hepatic colony-forming units in culture (H-CFU-C) were analyzed after images were captured and stitched using a Keyence BZ-X710 microscope and BZ-X Analyzer software (Keyence, Osaka, Japan) at day 3 or day 5 according to our reported methods [31, 32].

### qPCR

Total RNA was extracted from freshly isolated PCs and NPCs with Isogen (Nippon Gene, Tokyo, Japan). An amount of 1 μg RNA was converted to cDNA using a Revert Aid RT kit (Thermo Fisher Scientific), according to the manufacturer’s instructions. The quantitative polymerase chain reaction was performed in triplicate using the ABI TaqMan® Gene Expression Assays (Applied Biosystems, Foster City, CA, USA) on the ABI PRISM 7500 Real-Time PCR System (Applied Biosystems). The expression of the targeted genes was normalized to 18S as an endogenous control.

### Western blotting

Equal amounts of protein extracts of the cells were separated by electrophoresed in 12% SDS-PAGE gels and transferred onto the polyvinylidene difluoride membranes (Thermo Fisher Scientific). The membranes were blocked with 5% BSA and 0.1% Tween-20 in Tris-buffered saline for 1 h at room temperature, and then incubated overnight at 4 °C with the following primary antibodies: goat anti-galectin-1 (R&D), mouse anti-galectin-3 (Abcam), and rabbit anti-β-actin (Cell Signaling Technology, MA, USA). Membranes were washed; incubated with horseradish peroxidase-conjugated anti-goat IgG (R&D), anti-mouse IgG (Cell Signaling Technology), and anti-rabbit IgG (Cell Signaling Technology) secondary antibodies for 1 h at room temperature; and developed by ELC Western Blotting Detection Reagents (Amersham, GE Healthcare, UK). The blotted membranes were visualized using an ImageQuant LAS 4000 mini (GE Healthcare).

### Histology and immunohistochemistry

For hematoxylin and eosin (H&E) staining, liver tissues from sacrificed rats were fixed with 40 g/L neutral formaldehyde, dehydrated with ethanol and xylene, and followed by the standard paraffin-embedding procedure. The 4-μm-thick paraffin sections were cut and subjected to H&E staining.

For immunofluorescence analysis, cryostat sections or cultured cells were fixed in methanol: acetone (1:1) for 30 min at − 30 °C, and then blocked with 10% normal goat serum (Thermo Fisher Scientific) for 1 h at room temperature. The primary antibodies specifically against cytokeratin 19 (CK19; Progen, Heidelberg, Germany), OV-6 (R&D), CD44 (BD Biosciences, Franklin Lakes, NJ), CD133 (Abcam), EpCAM (Abcam), Ki67 (Abcam), desmin (Agilent, Santa Clara, CA, USA), Laminin (Sigma), SE-1 (IBL, Gunma, Japan), CD68 (Abcam), galectin-1 (R&D), or galectin-3 (R&D) were incubated at 4 °C overnight followed by washing three times with PBS. Secondary antibodies included Alexa Fluor 488, 555, or 647-conjugated secondary antibodies (Thermo Fisher Scientific) and were incubated at room temperature for 1 h. Nuclei were counterstained with 4′,6-diamidino-2-phenylindole (DAPI). The staining was visualized with a Zeiss AxioImager microscope (Carl Zeiss, Jena, Germany).

### Statistical analysis

GraphPad Prism v. 8.1.0 (GraphPad Software, San Diego, CA, USA) was used to analyze the data presented as mean ± SD (standard deviation) of at least three independent experiments. Welch’s *t* test was performed to compare the difference between the two groups. One-way ANOVA, followed by Bonferroni’s multiple comparisons test, was applied when more than two groups were analyzed. *P* values < 0.05 were considered significant.

## Results

### Hepatic stem/progenitor cells respond to liver injury

**Fig. 1:**
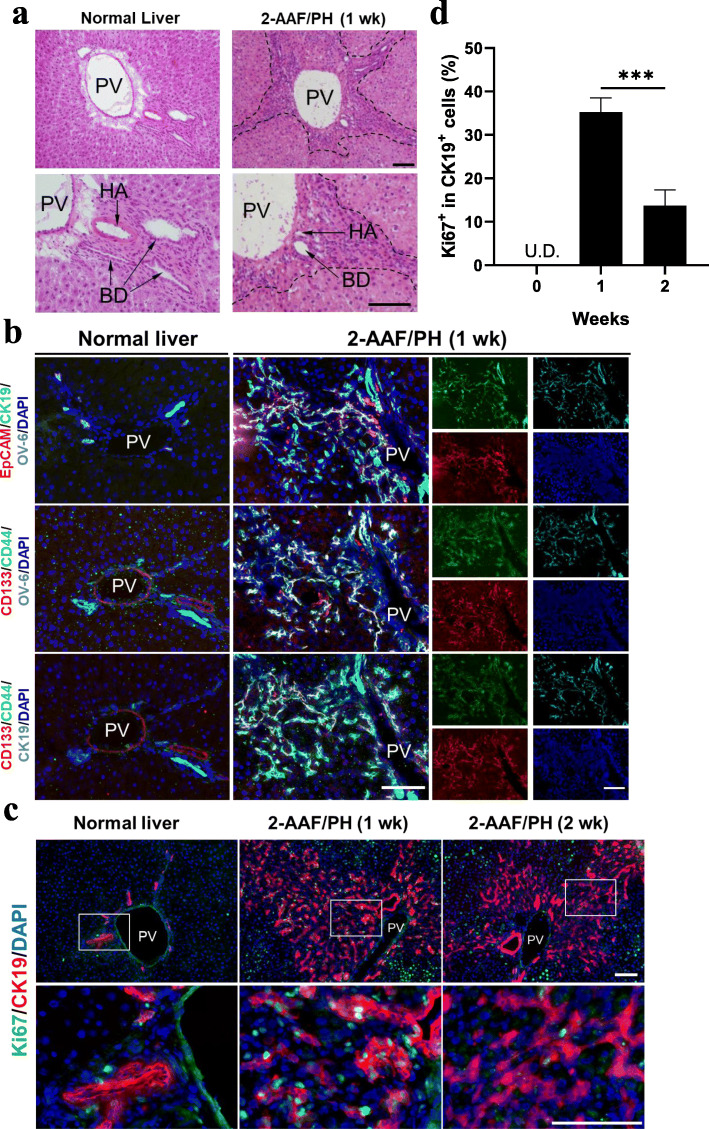
Hepatic stem/progenitor cells are induced following liver injury. **a** Hepatic oval cells (dotted area) were induced in 2-acetylaminofluorene plus 70% partial hepatectomy (2-AAF/PH)-treated liver (1 week and normal control, H&E stained). **b** Immunohistochemical co-localization of hepatic stem/progenitor related markers (CK19, OV-6, EpCAM, CD44, and CD133) in normal liver and 2-AAF/PH model liver (1 week). **c** Dual staining for CK19 and Ki67 at 0, 1, and 2 weeks after 2AAF/PH. **d** Quantification of **c** showed a significant elevation of Ki67^+^ proportion in CK19^+^ cells (*n* = 4). Nuclei were stained with DAPI. Scale bar, 100 μm. Data are shown as means ± SD. ^***^P < 0.001. PV, portal vein; BD, bile duct; HA, hepatic artery; U.D., undetectable

In a normal liver, ductular structures are exclusively restricted around the portal vein (PV). However, following induced liver injury, the activated ductal cells migrated from the periportal area and into the parenchyma (Fig. 1a). To characterize the phenotype of the activated cells in response to liver injury, immunofluorescence staining was performed to examine the expression of hepatic stem/progenitor related markers. It was revealed that most cells that expressing CK19, were also positive for OV-6, a definitive hepatic oval cell marker. Moreover, other stemness markers such as CD133, CD44, and EpCAM, all of which are rarely detected in normal liver, were also found co-expressed in OV-6^+^ and CK19^+^ cells (Fig. 1b). Furthermore, a significantly elevated proportion of proliferative cells (Ki67^+^) were observed periportally after liver injure (Fig. 1c), especially in the CK19^+^ cells, peaking at 1 week with a percentage of 35.2 ± 3.3% (Ki67^+^ in CK19^+^ cells), followed by a marked decrease at week 2 (Fig. 1d). These data indicated that the HSPCs were induced, enriched, and underwent an expansion in response to induced liver injury.

### Hepatic stellate cell recruitment during liver regeneration

**Fig. 2:**
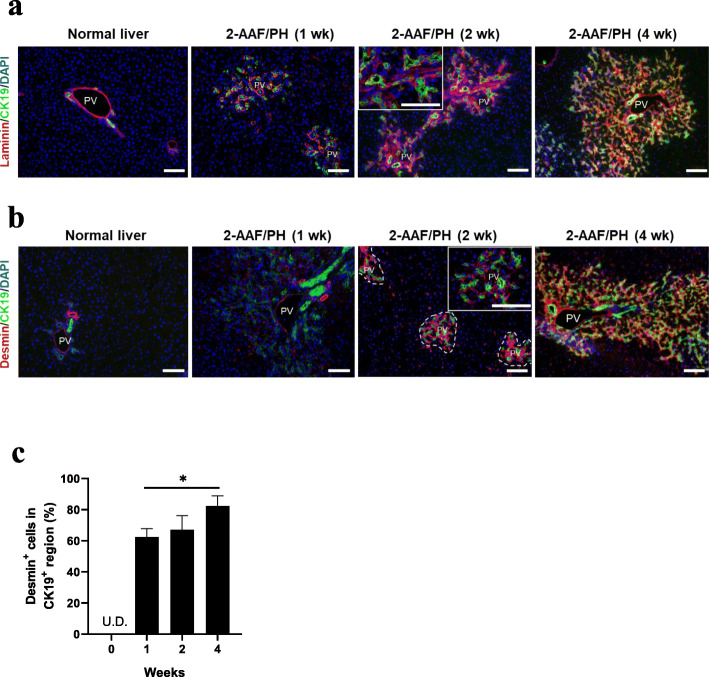
Hepatic stem cell niche emerges with the recruitment of hepatic stellate cells. **a** Dual staining of laminin and CK19 showed an expanding laminin sheath surrounding in the CK19^+^ periportal area during liver regeneration (weeks 0, 1, 2, and 4). **b** Localization of hepatic stellate cells was examined by co-staining of desmin and CK19. The dotted area (middle image) represents the periportal area enriched with CK19^+^ cells. **c** Statistical analysis of the percentage of desmin^+^ cells localized in the CK19^+^ region from week 0 to 4 (*n* = 4). Nuclei were stained with DAPI. Scale bar, 100 μm. Data are shown as means ± SD. ^*^P < 0.05. PV, portal vein; U.D., undetectable

Laminin, an essential ECM component in the hepatic stem cell niche, was found exclusively formed among the ductular structures surrounding in the periportal area and gradually radiated outwards following liver injury induction (Fig. 2a). To reveal the activation of HSCs in the niche, the localization and expansion of desmin^+^ cells were monitored over time. The number of activated HSCs, which are desmin-positive, notably increased in the regenerating liver compared to the normal liver; these cells expanded along with the CK19^+^ HSPCs (Fig. 2b). Moreover, the cells surrounding the CK19^+^ periportal area were markedly enriched compared to those in the non-periportal area. Additionally, the percentage of desmin^+^ cells in the CK19^+^ region increased from 64.7 ± 5.5% at week 1 to 82.3 ± 6.7% at week 4 (*P* < 0.05) (Fig. 2c). Thus, HSCs appeared to be co-activated with HSPCs during liver regeneration.

### Galectin upregulation accompanies liver regeneration

**Fig. 3:**
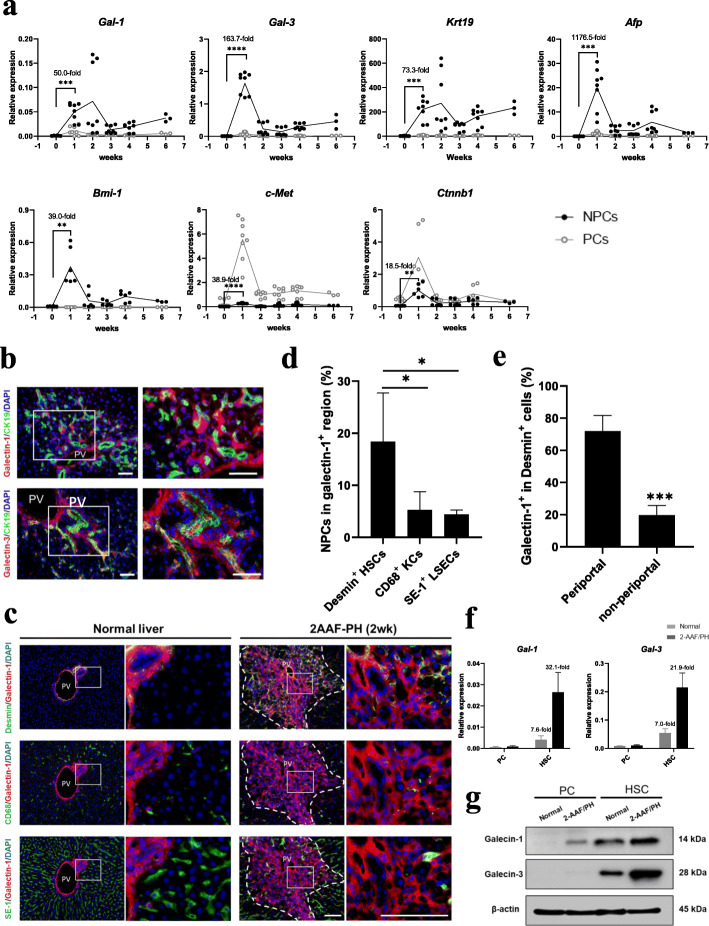
Galectin expression is enhanced during liver regeneration. **a** Dynamic genes expression changes of hepatic stem/progenitor related markers (*Gal-1*, *Gal-3*, *Krt19*, *Afp*, *Bmi-1*, *c-Met*, and *Ctnnb1*) in liver non-parenchymal cells (NPCs) and parenchymal cells (PCs) at weeks 0, 1, 2, 3, 4, and 6 following 2-AAF/PH (*n* = 6–8 for weeks 0, 1, 2, 3, and 4; *n* = 3 for week 6). The fold changes of genes expression in NPCs at week 1 (versus week 0) are shown. **b** Immunohistochemical detection of the co-localization of galectin-1 and galectin-3 with CK19 at week 2. **c** Co-localization of galectin-1 expression with desmin^+^ HSCs, CD68^+^ KCs, and SE-1^+^ LSECs was determined at week 2. The dotted area indicates the galectin-1 positive area. **d** Quantification of **c** showed markedly enriched HSCs in the galectin-1^+^ region (*n* = 4). **e** Comparison of the galectin-1^+^ proportion in desmin^+^ cells in periportal and non-periportal areas at week 2 (*n* = 4). **f**, **g** Gene (**f**) and protein expression level (**g**) of galectin-1 and galectin-3 in HSCs from normal and regenerating livers**.** The fold changes of gene expression between PC and HSC are shown (*n* = 3). Nuclei were stained with DAPI. Scale bar, 100 μm. Data are shown as means ± SD. ^*^P < 0.05, ^**^P < 0.01, ^***^P < 0.001, ^****^P < 0.0001. HSCs, hepatic stellate cells; KCs, Kupffer cells; LSECs, liver sinusoidal endothelial cells; PV, portal vein

To evaluate the dynamic changes of gene expression during liver regeneration, liver NPCs and PCs were isolated by perfusion and analyzed by qPCR analysis at weeks 0, 1, 2, 3, 4, and 6 after oval cell induction. Firstly, the successful isolation of PCs and NPCs were confirmed by their specific marker genes expression. It was revealed that the expression of PC marker *Alb* was 24.9-fold higher in PC than NPC fraction, while NPC markers *Vim*, *Stab2*, and *CD163* in NPC were 8.1-fold, 8.5-fold, and 9.2-fold higher than PC fraction, respectively (Additional file 1: Fig. S1a). Moreover, a 93.1 ± 3.0% purity in PC and 96.8 ± 2.3% purity in NPC were determined by microscopic examination (Additional file 1: Fig. S1b). Within the fractionated cells from the regenerative liver, HGF and WNT signaling associated genes, *c-Met*, and *Ctnnb1* were significantly induced in PCs and NPCs. In NPCs, *c-Met* expression increased 38.9-fold and *Ctnnb1* expression increased 18.5-fold at week 1 compared to week 0 (Fig. 3a)*.* More importantly, the selected stemness gene markers and *Gal-1* and -*3* were dominantly expressed in NPC rather than PC fraction. In NPCs, *Krt19* (73.3-fold increase), *Afp* (1176.5-fold increase)*,* and *Bmi-1* (39.0-fold increase), the genes expression involved in HSPCs induction and expansion were significantly elevated at week 1 compared to week 0. Meanwhile, galectins’ gene expression increased 50.0-fold (*Gal-1*) and 163.7-fold (*Gal-3*) at week 1, compared to week 0 (Fig. 3a). Along with the induction of gene expression, immunofluorescence imaging showed that galectin-1 and -3 were localized predominantly in the periportal area, especially enriched in and around the CK19^+^ HSPCs, with the minimal signal detected in the non-periportal area (Fig. 3b). To reveal the relationship between galectin expression and the recruitment of NPC components within the niche, the localization of desmin^+^ HSCs, SE-1^+^ liver sinusoidal endothelial cells, and CD68^+^ Kupffer cells were examined in the galectin-1^+^ region (Fig. 3c). A significantly higher enrichment was observed in HSCs compared to the other cell components (Fig. 3d). To identify the spatial expression characteristic of galectin-1 in HSCs, the correlation between HSC localization and incidence of galectin-1 positivity was investigated. As shown in Fig. 3e, galectin-1 exhibited greater expression in the activated HSCs localized in the periportal area than in those localized in the non-periportal area (72.1 ± 10.2% versus 19.5 ± 6.3%, *P* < 0.001). Furthermore, the elevation of gene and protein expression for galectin-1 and -3 were confirmed in HSCs during liver regeneration by qPCR and western blotting (Fig. 3f, g). These results suggest a potential role for galectins in the activation of HSCs within the hepatic stem cell niche.

### HSCs promote the expansion of hepatic stem cells through a galectin-mediated pathway

**Fig. 4:**
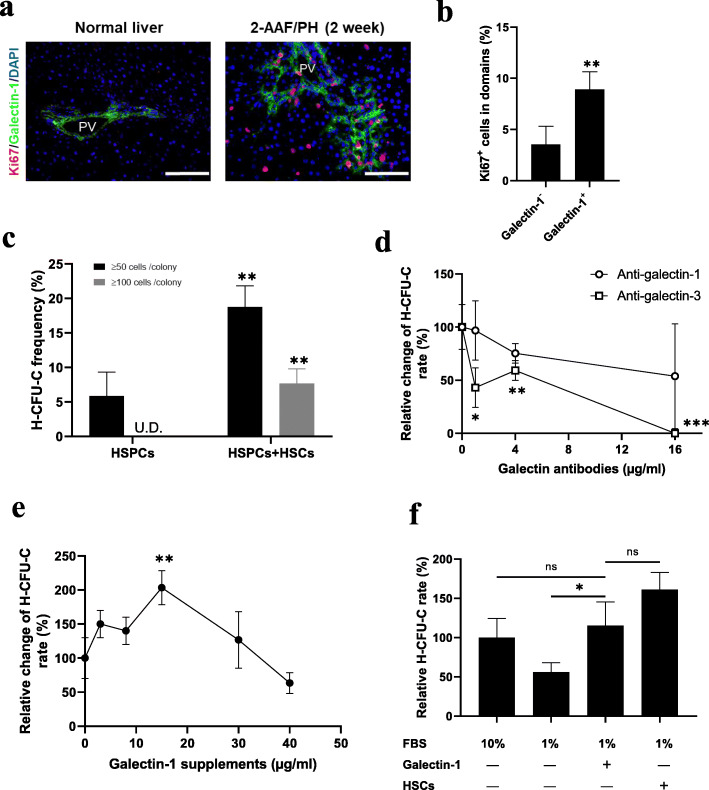
Galectins mediate hepatic stem cell expansion. **a** Co-localization of galectin-1 and Ki67 at 2 weeks after 2-AAF/PH treatment. Scale bar: 100 μm. **b** Proportion of the Ki67^+^ cells enriched in galectin-1 positive and negative domains. **c** Frequency of H-CFU-C with size over 50 cells/colony and 100 cells/colony were compared with or without HSCs co-culture at day 5 (*n* = 4). **d**, **e** Relative change of H-CFU-C rate under supplements of different concentrations of anti-galectin-1/anti-galectin-3 antibodies (**d**), or under supplements of recombinant mouse galectin-1 with different concentrations (**e**). Mean value of the no treatment samples was set as reference (*n* = 3–5). **f** Relative H-CFU-C rate in the presence of 10% FBS, 1% FBS, and 1% FBS with supplements of recombinant mouse galectin-1 (15 μg/ml) or with HSCs co-culture (n = 4). Nuclei were stained with DAPI. Data are shown as means ± SD. ^*^P < 0.05, ^**^P < 0.01, ^***^P < 0.001. ns, no significance; PV, portal vein; HSCs, hepatic stellate cells; HSPCs, hepatic stem/progenitor cells; H-CFU-C, hepatic colony-forming units in culture

To evaluate whether the galectins were engaged in promoting the cell expansion within the niche, galectin-1 and Ki67 expression was examined. As shown in Fig. 4a, most Ki67^+^ proliferative cells were found enriched around the galectin-1 positive domains, and the proportion was significantly higher than in galectin-1 negative domains (Fig. 4b). To further identify whether galectins directly and/or indirectly mediated the promotive function of HSCs on hepatic stem cells expansion, c-Kit^−^CD29^+^CD49f^+/low^CD45^−^ Ter-119^−^ hepatic stem cells were isolated and employed as an effective target (Additional file 2: Fig. S2), as these cells possessed extensive self-renewal and clonal expansion capabilities. When the hepatic stem cells were co-cultured with HSCs in the transwell system (Additional file 2: Fig. S2), the frequency of hepatic colony-forming units in culture (H-CFU-C, > 50 cells/colony) was found to be significantly elevated. Furthermore, the enlarged colonies were also observed (> 100 cells/colony) (Fig. 4c). However, the addition of the anti-galectin-1 or -3 blocking antibody impaired the colony-forming efficiency (Fig. 4d). The addition of recombinant mouse galectin-1 was found to promote H-CFU-C frequency in a concentration-dependent manner at concentrations below 30 μg/ml. Further increasing the galectin-1 concentration impaired the colony formation (Fig. 4e). Importantly, the addition of an optimized concentration (15 μg/ml) of galectin-1 alone significantly enhanced the H-CFU-C frequency in low FBS (1%) condition, reaching a level comparable to that in the control with 10% FBS, although slightly lower than that in co-culture with HSCs (no significance) (Fig. 4f). These data demonstrated the galectins might serve essential signaling components mediating the promotive function of HSCs on hepatic stem cell expansion.

### Galectins loss of function in HSCs impaired the clonal expansion of hepatic stem cells

**Fig. 5:**
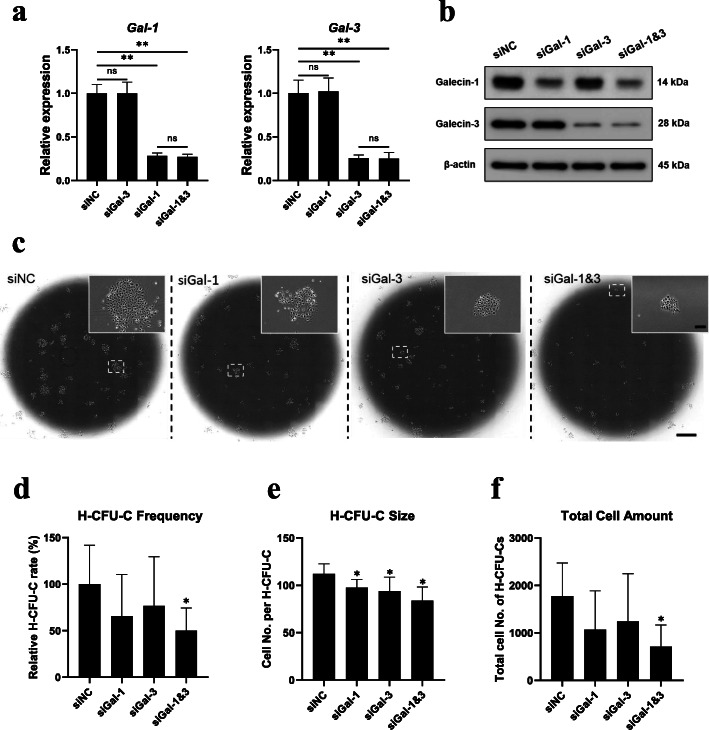
Inhibition of galectins expression in HSCs impaired the clonal expansion of co-cultured hepatic stem cells. **a**, **b** Gene and protein expression of galectin-1 and -3 in HSCs by qPCR (**a**) and western blotting (**b**) analysis 72 h after the transfection with siNC (negative control), siGal-1, siGal-3, and siGal-1&3. **c**–**f** Representative images of the clonal expansion of hepatic stem cells (**c**), H-CFU-C frequency (**d**), H-CFU-C size (**e**), and total cell amount (**f**) after 3 days co-culture with HSCs pre-treated with siNC, siGal-1, siGal-3, and siGal-1&3 (*n* = 4, compared with siNC). Scale bar, 200 μm (**c**, upper) and 2 mm (**c**, lower). Data are shown as means ± SD. ^*^P < 0.05, ^**^P < 0.01. ns, no significance; HSCs, hepatic stellate cells; H-CFU-C, hepatic colony-forming units in culture

To determine whether inhibition of galectin expression in HSCs could impair their promotive effect on the clonal expansion of hepatic stem cells, HSCs were transfected with siRNA against galectin-1 (siGal-1) and/or galectin-3 (siGal-3) ahead of co-culture. The transfection of siGal-1 and siGal-3 resulted in 71.7% and 74.3% knockdown of *Gal-1* and *Gal-3* gene expression, respectively, when compared to transfection control (siNC), with no obvious interfere to the gene expression of each other. Moreover, double knockdown with siGal-1&3 led to a compositive effect of a single treatment of siGal-1 or siGal-3 but did not further enhance the knockdown efficiency (Fig. 5a). The marked inhibition of galectin-1 and galectin-3 was also confirmed in protein expression by western blotting (Fig. 5b). Interestingly, the knockdown of galectins did not induce altered cell morphology (Additional file 3: Fig. S3a) and the gene expression of *Des*, *Acta2*, and *Col1a1*, which are closely associated with HSCs activation and fibrogenic, remained unchanged (Additional file 3: Fig. S3b). When co-cultured with hepatic stem cells in low FBS (1%) condition, knockdown of either *Gal-1* or *Gal-3* led into a significant reduction of H-CFU-C size (Cell No. per H-CFU-C) to 87.1% and 83.8%, respectively, when compared to siNC, while double knockdown of galectin-1 and -3 further impaired the H-CFU-C size to 74.9% (Fig. 5e). Moreover, the markedly suppressed H-CFU-C frequency (Fig. 5d) and total cell amount in H-CFU-Cs (Fig. 5f) were uncovered under double knockdown treatment. These findings indicated that HSCs-secreted galectins might synergistically promote the colony-forming efficiency and the expansion of hepatic stem cells, suggesting a supportive role in maintaining their self-renewal potential.

## Discussion

Clarification of the hepatic stem cell niche composition and function in the healthy and diseased liver is critical to understand liver regeneration, cellular and organoid transplantation, and carcinogenesis, as well as to develop therapeutic strategies for treating liver diseases. Within the niche, both cell-cell and cell-matrix signaling are believed to be closely associated with the induction of HSPCs. Among them, the coordinative interaction between HSPCs and HSCs has been particularly emphasized, as their co-activation was identified during liver regeneration [33, 34] and liver fibrogenesis [35].

The well-known signaling pathways, such as Notch and canonical WNT, which are mediated by HSCs and macrophages, have been proved to influence HSPCs expansion, differentiation, and migration [9, 10]. In recent years, the β-galactoside-binding lectin family members, especially galectin-1 and galectin-3, were revealed to play important roles in HSPCs expansion and liver regeneration [21, 22]. However, little is known regarding the role of galectins in cross-talking between HSPCs and other niche components.

In this study, for the sake of establishing a stable hepatic stem cell niche, we utilized the rat hepatic oval cell model, where chemical inhibition of hepatocyte proliferation by 2-acetylaminofluorene combined with partial hepatectomy induces a robust HSPCs response [7]. In accordance with previous studies, the CK19^+^OV-6^+^ HSPCs were induced together with the formation of a laminin sheath and the recruitment of HSCs.

To identify the dynamic induction of galectins during liver regeneration, *Gal-1* and *Gal-3* expression were monitored in a time-dependent manner. We found that their expression showed a pattern similar to that of genes that reportedly maintain the stemness of HSPCs, such as *Afp*, *Krt19,* and *Bmi-1*, and genes involved in WNT and HGF signaling pathways, such as *Ctnnb1* and *c-Met*, suggesting a coordinated interaction between galectins and hepatic stem cell niche signaling. Moreover, the induction of *Gal-1* and *Gal--3* were found dramatically higher in NPCs than PCs. Due to the limitation of the current separation protocol, NPC contamination in the isolated PC pellets cannot be excluded. To address whether the hepatocytes expressed galectins in this model, individual cells were examined in the liver sections. Galectin distribution in liver sections from the rat model was rarely observed outside the periportal area, especially in hepatocytes with classic polygonal appearance. Thus, the induction of galectin expression appeared to be restricted to NPCs. Furthermore, considering that in the current model, hepatocyte proliferation was inhibited by 2-AAF, it remains to be elucidated if galectins are involved in the replication of hepatocytes following acute injury. Numerous galectin-expressing cells were enriched around the periportal area in the regenerating liver, which was in accordance with the previous report in a choline-deficient ethionine mouse model [21]. Importantly, the expression of galectins was found not restricted to CK19^+^ cells, but also in CK19^−^ neighboring cells, which suggested they may also be involved in activation of other cell types within the niche, and mediate the cell-cell interactions. Regarding the important role of HSCs, liver sinusoidal endothelial cells, and Kupffer cells within the hepatic stem cell niche [11], their localization was examined in the regenerating liver. Interestingly, only activated HSCs were specifically enriched around the galectin-1 positive regions, while sinusoidal endothelial cells and Kupffer cells seemed randomly distributed along the liver lobule. Thus, we hypothesized the galectins may engage in HSCs activation, as well as its interaction with HSPCs. Since it was reported that galectin-1 but not galectin-3 could specifically promote the migration of HSCs [24], in our study, we specially examined the correlations between HSCs location and their galectin-1 expression in the regenerating liver. The observation that galectin-1 expression was markedly enriched in activated HSCs localized within the CK19^+^ periportal regions suggested that galectin-1 might mediate migration of HSCs from non-periportal regions toward the hepatic stem cell niche in response to liver injury. Meanwhile, the expression of galectin-1 and -3 in HSCs was sustained even after they were isolated and cultured, which warrants further investigation whether HSCs promote the expansion of HSPCs via a process involving galectin-mediated regulation in vitro. In the transwell culture, our results revealed that co-culture with HSCs, as well as exposure to recombinant galectin protein alone, significantly promoted the expansion of c-Kit^−^CD29^+^CD49f^+/low^CD45^−^ Ter-119^−^ hepatic stem cells, as verified by enhanced colony-forming frequency and enlarged colony size. The inhibition of colony formation by anti-galectin antibodies further suggested the potential role of galectins in the HSC-HPSC signaling. Furthermore, the knockdown of galectin-1 and galectin-3 in HSCs significantly impaired their promotive effect on the clonal expansion of hepatic stem cells, as shown with reduced H-CFU-C efficiency, demonstrating HSCs-derived galectins may serve critical niche signaling activities involved in the cross-talking between activated HSCs and HSPCs. It may be partially explained by the independent observations that galectins can directly and/or indirectly activate cyclin D1 in the nucleus, hyperactivate the WNT signaling pathway, and eventually promote HSPCs expansion [36, 37]. Moreover, galectins may also regulate lysophosphatidic acid (LPA) signaling by modulating the expression of autotaxin, which is responsible for its synthesis [38]. Since LPA could activate β-catenin and subsequently promote stem cell proliferation and self-renewal [39], a potential galectin-autotaxin-LPA axis is suggested to underlie the niche signaling between HSCs and HSPCs. Interestingly, β-catenin has also been identified to be critical for mesenchymal cell activation and fibrosis in vivo [40]; thus, the same axis may also be responsible for liver fibrosis initiated from HSCs activation. Significantly reduced liver fibrosis had been achieved following inhibition of ATX and/or LPA receptor 1 [41, 42], suggesting potential antifibrotic strategies targeting galectin-autotaxin-LPA axis. Besides, the potential modulations regarding other signaling pathways cannot be fully excluded. Moreover, it was found that the effect of galectin was biphasic and concentration-dependent. A limited range of concentrations below 30 μg/ml could promote the expansion, while higher concentrations led to the opposite results. Besides, a 90% knockdown of *Gal-3* was reported to inhibit myofibroblast activation and procollagen expression [17], while in this study, a near 70% knockdown of either *Gal-1* or *Gal-3* seemed not effectively induce the suppression. It may reflect the multifunctional roles of galectins as previously validated [43, 44] and also highlight their notable roles in hepatic stem cell niche responding to liver injury-induced regeneration, liver fibrosis progression, as well as hepatic carcinogenesis.

To the best of our knowledge, this is the first study demonstrating the possible role played by galectins in modulating HSCs activation and their promotive effect on HSPCs expansion during liver regeneration. However, the detailed mechanism(s) of action of galectins in niche signaling were not addressed in the current study. Further investigations are warranted to elucidate their detailed involvements in pathway signaling regarding liver regeneration and disease progression signaling, as well as their role in regulating other cellular and matrix niche components. Comprehensive clarification of their biphasic modulation manner within the hepatic stem cell niche may open new perspectives in developing novel galectin-targeted therapeutic strategies for liver diseases.

## Conclusions

The results of this study showed, for the first time, that galectins are involved in the interactions between HSCs and HSPCs, particularly in mediating HSPC expansion. This study provides evidence that galectins may serve as important signaling components in the hepatic stem cell niche.

## Supplementary information

**Additional file 1: Fig. S1** Confirmation and purity examination of isolated PC and NPC fractions after liver perfusion. **a** Gene expression of *Alb*, *Vim*, *Stab2*, and *CD163* in LC, PC, and NPC fractions by qPCR. Fold change between PC and NPC was indicated (*n* = 3). **b**. Representative images of PCs and NPCs 24 h after isolation. Scale bar: 100 μm. Data are shown as means ± SD. LC: liver cell; PC: parenchymal cell; NPC: non-parenchymal cell.

**Additional file 2: Fig. S2** Setup of transwell co-culture for HSCs and hepatic stem cells. HSCs were seeded on the membrane of transwell inserts with a 0.4 mm pore size. c-Kit^−^CD29^+^CD49f^+/low^CD45^−^Ter-119^−^ hepatic stem cells were sorted by flow cytometry and seeded on 6-well plate pre-coated with type IV collagen. Co-culture for HSCs and hepatic stem cells was set up 1 day later. HSCs: hepatic stellate cells.

**Additional file 3: Fig. S3** Knockdown of galectins did not disrupt the expression of fibrogenesis-related markers in HSCs. **a**, **b** Cell morphology (**a**) and gene expression of *Des*, *Acta2*, and *Col1a1* (**b**) in HSCs 72 h after treatment with siNC, siGal-1, siGal-3, and siGal-1&3. Scale bar: 200 μm. Data are shown as means ± SD. *n* = 3. HSCs: hepatic stellate cells.

## Data Availability

All data supporting the findings of this study are available.

## Abbreviations

HSPCs: Hepatic stem/progenitor cells
HSCs: Hepatic stellate cells
2-AAF: 2-acetylaminofluorene
PH: Partial hepatectomy
CK19: Cytokeratin 19
ECM: Extracellular matrix
PCs: Parenchymal cells
NPCs: Non-parenchymal cells
H-CFU-C: Hepatic colony-forming units in culture
PV: Portal vein
BD: Bile duct
HA: Hepatic artery
H&E: Hematoxylin and eosin
LPA: Lysophosphatidic acid
