# Supplementary figures and images for: Hepatic stellate cells contribute to liver regeneration through galectins in hepatic stem cell niche
Source: Stem Cell Res Ther. 2020 Sep 29;11:425. doi: 10.1186/s13287-020-01942-x (PMC7526193; doi:10.1186/s13287-020-01942-x)

## Slide 1
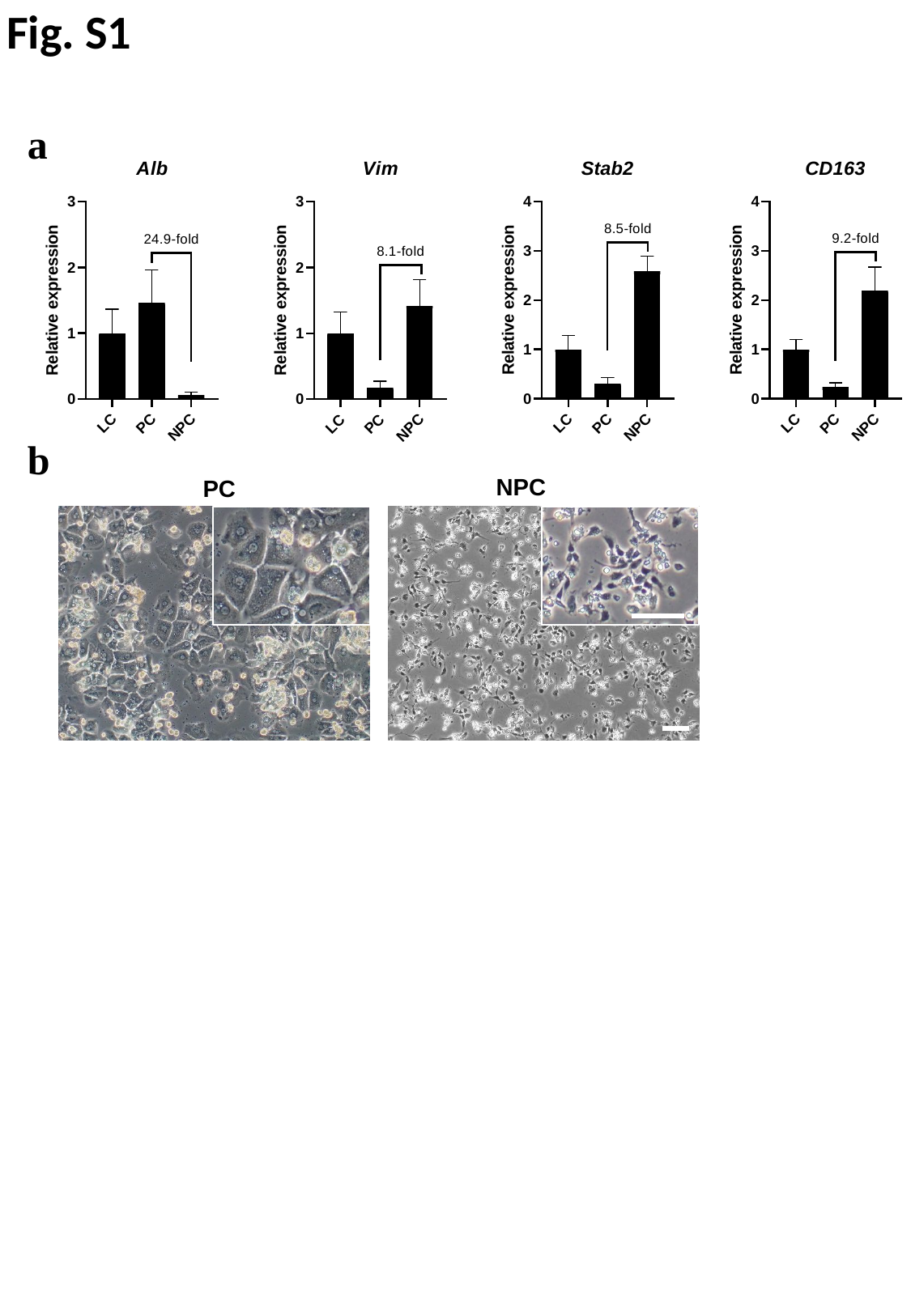

Fig. S1
a
b
NPC
PC

Supplement: Supplementary file 1 — Additional file 1: Fig. S1 Confirmation and purity examination of isolated PC and NPC fractions after liver perfusion. a Gene expression of Alb, Vim, Stab2, and CD163 in LC, PC, and NPC fractions by qPCR. Fold change between PC and NPC was indicated (n = 3). b. Representative images of PCs and NPCs 24 h after isolation. Scale bar: 100 μm. Data are shown as means ± SD. LC: liver cell; PC: parenchymal cell; NPC: non-parenchymal cell. [file 13287_2020_1942_MOESM1_ESM.pptx]

## Slide 1
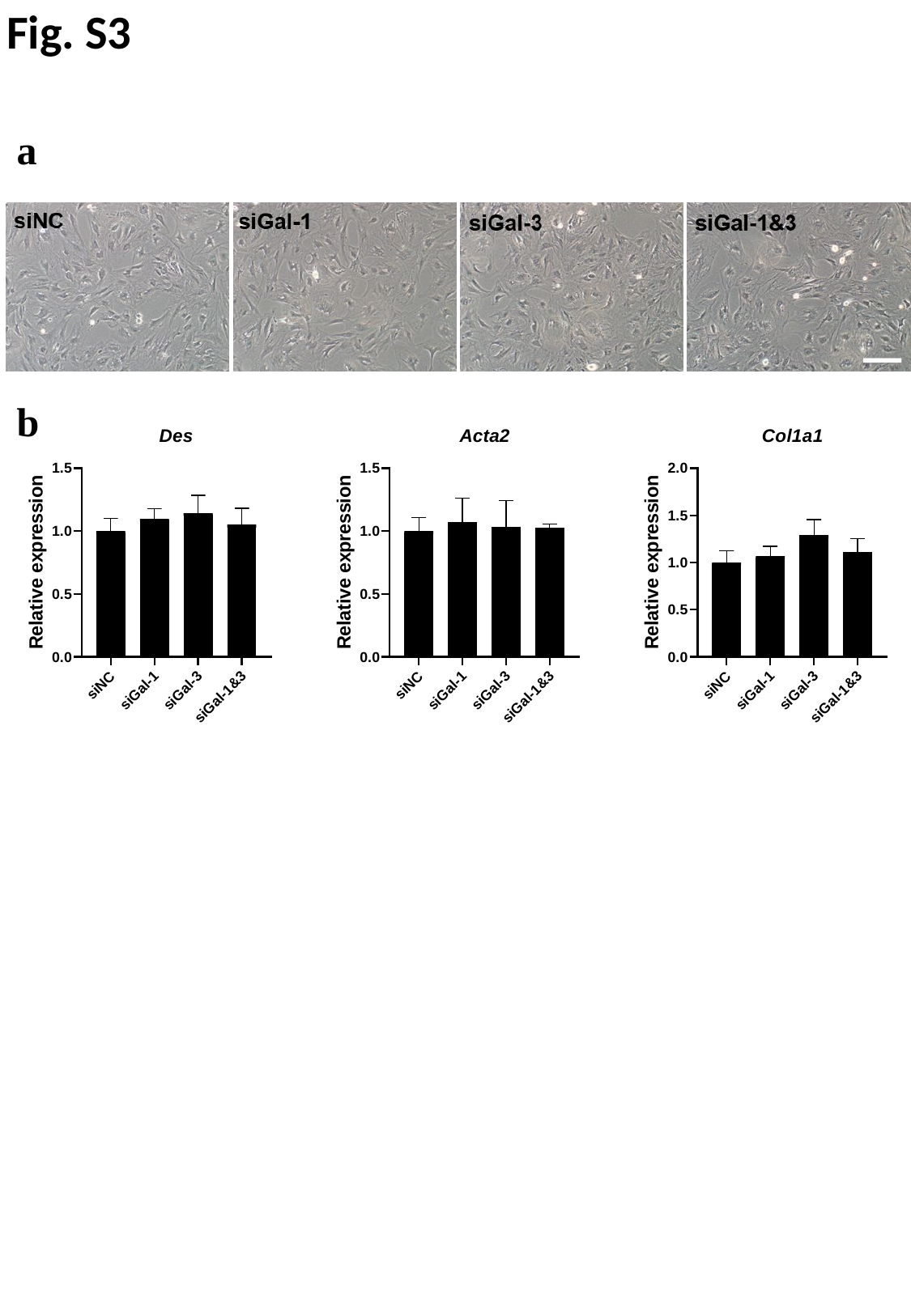

Fig. S3
a
b

Supplement: Supplementary file 3 — Additional file 3: Fig. S3 Knockdown of galectins did not disrupt the expression of fibrogenesis-related markers in HSCs. a, b Cell morphology (a) and gene expression of Des, Acta2, and Col1a1 (b) in HSCs 72 h after treatment with siNC, siGal-1, siGal-3, and siGal-1&3. Scale bar: 200 μm. Data are shown as means ± SD. n = 3. HSCs: hepatic stellate cells. [file 13287_2020_1942_MOESM3_ESM.pptx]
