# Supplementary material for: Hepatic stellate cells contribute to liver regeneration through galectins in hepatic stem cell niche
Source: Stem Cell Res Ther. 2020 Sep 29;11:425. doi: 10.1186/s13287-020-01942-x (PMC7526193; doi:10.1186/s13287-020-01942-x)
Supplement: Supplementary file 2 — Additional file 2: Fig. S2 Setup of transwell co-culture for HSCs and hepatic stem cells. HSCs were seeded on the membrane of transwell inserts with a 0.4 mm pore size. c-Kit−CD29+CD49f+/lowCD45−Ter-119− hepatic stem cells were sorted by flow cytometry and seeded on 6-well plate pre-coated with type IV collagen. Co-culture for HSCs and hepatic stem cells was set up 1 day later. HSCs: hepatic stellate cells. [file 13287_2020_1942_MOESM2_ESM.pptx]

## Slide 1
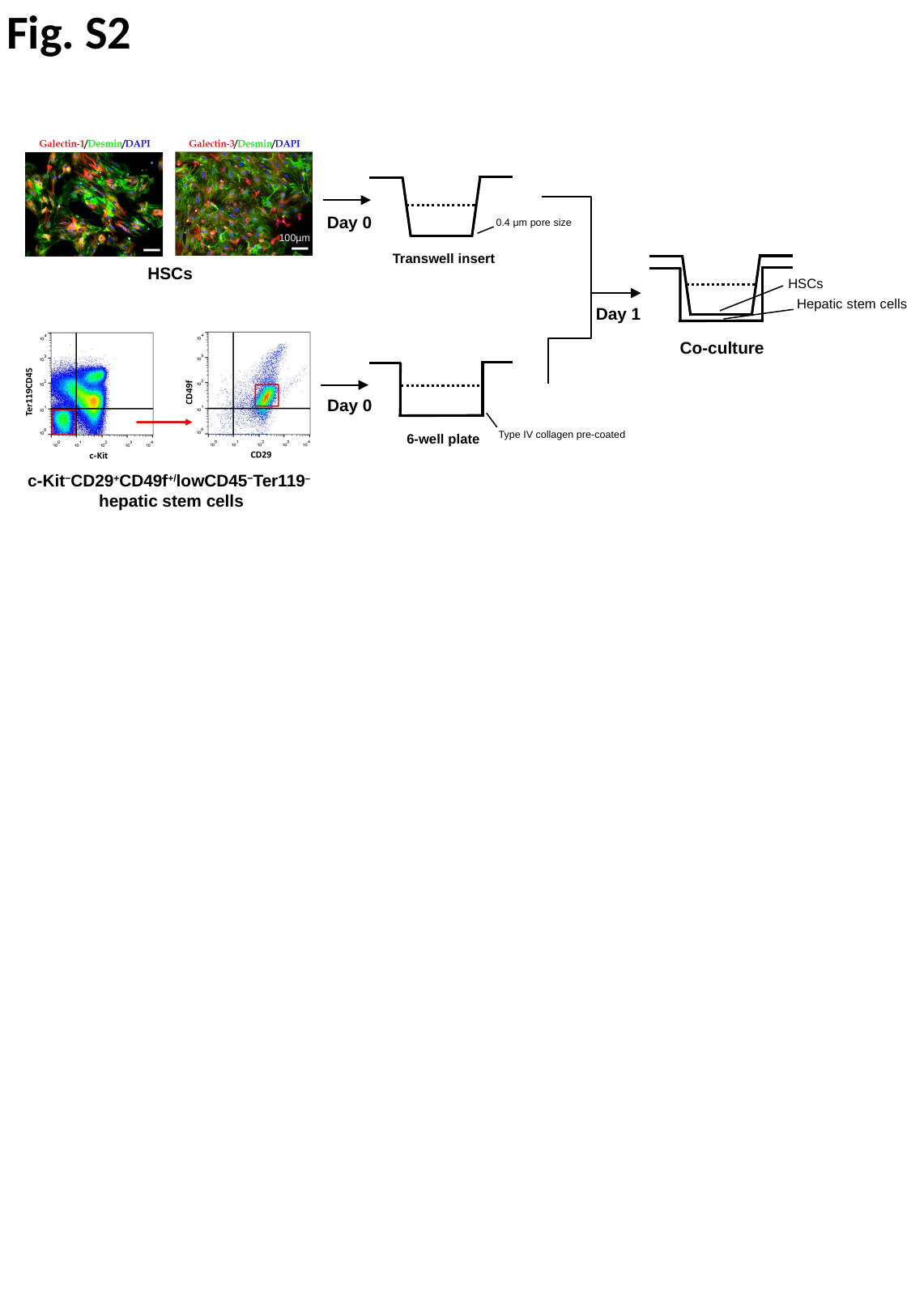

Fig. S2
Day 0
0.4 μm pore size
100µm
Transwell insert
HSCs
HSCs
Hepatic stem cells
Day 1
Co-culture
Day 0
Type IV collagen pre-coated
6-well plate
c-Kit−CD29+CD49f+/lowCD45−Ter119–
hepatic stem cells
